# Brain metastases in gastroesophageal cancers—an underestimated complication

**DOI:** 10.1007/s10120-021-01219-z

**Published:** 2021-07-23

**Authors:** Marius Brunner, Dominik Soll, Kathrin Adler, André Sasse, Ute König, Ardian Mekolli, Kristina Lowes, Johanna Reinecke, Volker Ellenrieder, Alexander König

**Affiliations:** 1 Department of Gastroenterology, Gastrointestinal Oncology and Endocrinology, University Medical Center Göttingen, Göttingen, Germany; 2 Department of Endocrinology and Metabolism, Charité - Universitätsmedizin Berlin, Berlin, Germany; 3 Department of Haematology and Oncology, University Medical Center Göttingen, Göttingen, Germany

**Keywords:** Gastric cancer, Esophageal cancer, GEC, Brain metastases, Incidence, Prognosis, Screening

## Abstract

**Background:**

Brain metastases represent a severe complication in many gastrointestinal malignancies especially those arising from the upper gastrointestinal tract, including cancer of the esophagus, gastroesophageal junction, and stomach (GEC). However, there is little knowledge about the onset or potential risk factors for brain metastases (BRMs) in upper gastrointestinal cancers resulting in a lack of screening guidelines for BRMs.

**Methods:**

We analyzed 827 patients from our cancer registry suffering from gastroesophageal cancer (GEC) and treated at the University Medical Center Göttingen between January 2013 and December 2019 for the presence of BRMs.

**Results:**

From 827 patients with GEC we found 54 patients with BRMs, resulting in an incidence of 6.5%. BRMs are more frequent in male patients (90.74% vs 9.26%, *p* = 0.0051) and in adenocarcinomas (90.74% vs 9.26%, *p* = 0.0117). Mean duration for the onset of BRMs from initial cancer diagnoses was 20.9 months in limited disease (curative approach) and 9.3 months in advanced disease (palliative approach) (*p* = 0.0026). However, early detection of BRMs is a prognostic factor since patients with successful resection of BRMs have a better prognosis compared to those with unresectable BRMs (5.93 vs 2.07 months, *p* = 0.0091).

**Conclusion:**

In this single-center retrospective study, brain metastases (BRMs) occur with a high frequency (6.5%) in gastroesophageal cancer (GEC), significantly more often in male patients and adenocarcinomas. Since survival of these patients considerably correlates with successful BRMs resection, our observations propose further prospective trails to validate our hypothesis and ultimately the implementation of routine screening procedures to detect asymptomatic brain metastases.

## Introduction

Gastroesophageal cancer (GEC) comprises epithelial malignancies of the esophagus, the gastroesophageal junction and the stomach. In the esophagus, carcinomas can emerge as either squamous cell carcinoma or adenocarcinoma (Sievert type I cancer), whereas carcinomas from the gastroesophageal junction (Sievert type II and III cancer) and the stomach are mostly adenocarcinomas. Due to its high incidence [1, 2] and poor prognosis, GEC represents one of the major challenges in gastrointestinal oncology [3]. Despite of major achievements in diagnostics, patients are often diagnosed at advanced stages (locally irresectable or metastatic disease) when curative surgery is no longer an option. One major reason for the poor prognosis of GEC is the early onset of distant metastases, which are mostly located in the liver, lungs, bones, and in the peritoneum [4]. Due to the metastatic pattern, imaging in tumor surveillance or therapy monitoring usually focuses on these four organs in particular.

In patients with advanced GEC (locally irresectable or metastatic disease), systemic chemotherapy usually represents the treatment of choice. Older but still widely used therapeutic regimes demonstrate mediocre antitumor activity with increased toxicity and corresponding short patient survival of approximately 12 months [5]. A platinum-based doublet chemotherapy with 5-fluorouracil/folinic acid or even a triplet chemotherapy with the further addition of docetaxel are frequently used first-line regimens in advanced GEC [6, 7]. Even after failure of platin-based first-line chemotherapy, patients often have an acceptable health status that allows for second-line or third-line treatment. Higher lines of therapy usually include vascular endothelial growth factor receptor (VEGFR) inhibition as an important therapeutic strategy [8]. However, recent studies have investigated to potential role of immune checkpoint inhibitors in treatment of advanced GECs in different treatment lines, creating even more treatment possibilities for patients with advanced GEC [9].

The combination of more effective chemotherapy protocols, including immune checkpoint inhibitors, prolongs patient survival and reduces tumor cell burden in major target organs for distant metastases. However, the more effective systemic treatment results in a higher incidence of new tumor-associated complications, such as long-term toxicity or the occurrence of metastases in organs where classical systemic treatment is less effective.

This includes above all the human brain, and more than 70,000 new cases of brain metastases were diagnosed in the United States in 2007 [10]. The vast majority of intracerebral metastases result from melanomas and adenocarcinomas of the breast and lung [10], where routine cerebral MRI imaging is highly recommended due to the high incidence of brain metastases in approximately 30% at the time of initial diagnosis [11]. However, this makes lung carcinoma rather exceptional and brain metastases (BRM) are usually diagnosed in advanced stages of malignant diseases when cerebral imaging is initiated due to new onset of neurological symptoms. Importantly, however, treatment options are then often limited and prognosis of patients is poor especially when resection or stereotactic radiation is no longer possible [11, 12]. Early detection of brain metastases is therefore of great clinical relevance for their effective treatment. In GEC, our understanding of both the incidence and clinical relevance of brain metastases is very limited [13, 14]. So far, only few retrospective analyses have been published including data from the Surveillance, Epidemiology, and End Results (SEER) program. A low incidence of brain metastases with 1.7% of patients with esophageal cancer and 0.6% of patients with gastric cancer within the last 25 years is reported [15, 16]. These reports are usually lacking relevant details of brain metastases, such as timeframe of onset or treatment strategies.

In the present work, we analyzed the cancer registry program of the Goettingen Comprehensive Cancer Center (G-CCC) for detection of brain metastases in patients with carcinomas of the upper gastrointestinal tract.

## Materials and methods

**Fig. 1:**
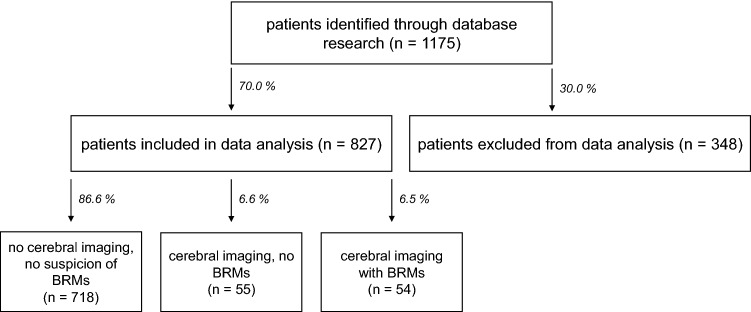
Flow chart of data selection. Flow chart demonstrating data base analysis and selection of patients with brain metastases (BRMs) resulting from gastroesophageal cancer. Primary inclusion criteria were a corresponding ICD-10 diagnosis (C15.x or C16.x) and hospital contact between 2013 and 2019. Exclusion criteria were a primary diagnosis before 2008 (insufficient data availability), history of other malignancies, other pathologies (GIST, lymphomas, gastric metaplasia, intestinal metaplasia), endoscopically removed tumors and patients with insufficient data and initial diagnosis after 2008

We screened the database of the Comprehensive Cancer Center at the University Medical Center Goettingen (Goettingen, Germany) for patients with ICD-10 diagnosis of C15.x and C16.x between 2013 and 2019. After applying the exclusion criteria (primary diagnosis before 2008, insufficient data, and other malignancies of the esophagus and stomach like lymphomas or gastrointestinal stroma tumors), we identified 827 patients with either squamous cell carcinoma or adenocarcinoma of the esophagus or adenocarcinoma of the gastroesophageal junction and the stomach that were treated between January 2013 and December 2019 (Fig. 1). Unless patients described symptoms possibly relating to BRMs, brain imaging was not routinely performed at the time of diagnosis or during the course of disease.

### Treatment and follow-up strategy

Patients were treated in accordance with the current German and European guidelines [17–20]. All patients were discussed in certified tumor boards of the Comprehensive Cancer Center (CCC). After reviewing all findings and reports, an interdisciplinary team of experts composed of experts from gastroenterology, medical oncology, thoracic and visceral surgery, radiation oncology, radiology and pathology, defined a treatment strategy for each patient according to national and international treatment recommendations. Patients were followed at 3- to 12-month intervals for up to 5 years during or after treatment. Cerebral imaging by CT or MRI scan was performed only in patients with suspected brain metastases indicated by neurological symptoms. Brain metastases were assumed when a distinct mass was identified by imaging or was determined by histological analysis of biopsy or resection. Treatment strategies for brain metastases were discussed at a multidisciplinary tumor board and defined by experts in neurosurgery and radiooncology. For all patients, we collected data for gender, histology and initial tumor stadium to determine significant risk factors for the development of cerebral metastases (Table 1).

**Table 1:** Clinical characteristics of analyzed patient collective. Patients without suspicion for brain metastases comprises both patients who had cerebral imaging without diagnosis of possible metastases and patients who did not have any cerebral imaging due to missing clinical signs

	Patients with brain metastases in cerebral imaging	Patients withouth suspicion for brain metastases	Significance
Gender			*p* = 0.0051
Male	49	572	
Female	5	201	
Strategy at initial diagnosis			*p* = 0.0201
Limited disease (resectable)	30	551	
Advanced disease (non-resectable)	24	222	
Histology			*p* = 0.0117
Adenocarcinoma	49	590	
Squamous cell carcinoma	5	183	

### Statistics

We used descriptive statistics by frequency (%) to summarize patient characteristics. We conducted *t* tests (Fisher’s exact test) to evaluate the significance of two different groups. A *p*-value of *p* < 0.05 was interpreted as a significant difference between two groups. The relative risk (RR) was calculated with the Koopman asymptotic score. The overall survival was determined as time from initial diagnosis to death caused by any cause. Furthermore, we displayed overall survival by Kaplan–Meier-curves. We used Log-rank (Mantel–Cox) tests to determine significance of survival curves. For all statistical analyses and graphic illustrations, we utilized either Graphpad Prism 9.1 (GraphPad Software, Inc., San Diego, CA) or R (R Core Team, Version 4.0.4).

## Results

**Fig. 2:**
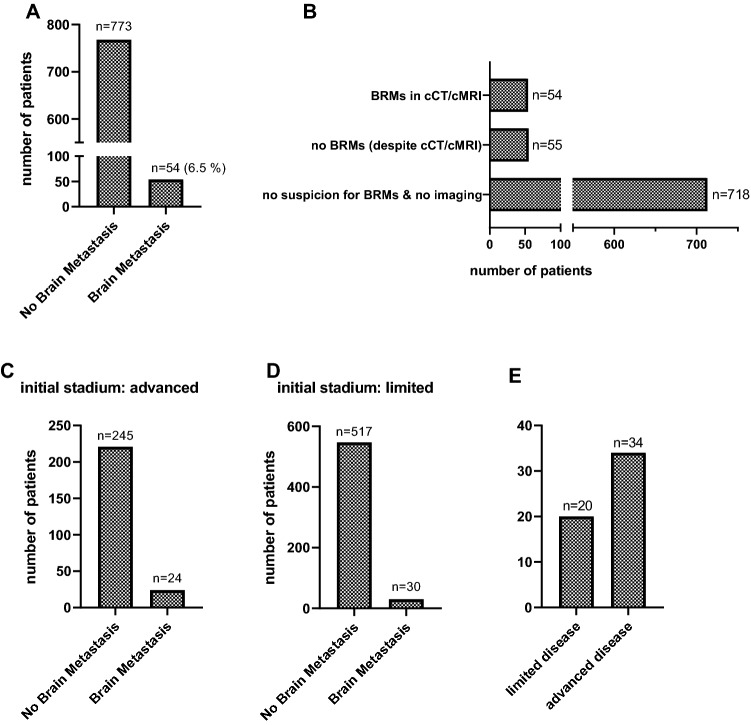
Incidence of brain metastases (BRMs). **A** Incidence of BRMs in patients with cancer of the stomach and esophagus (GEC). 828 patient data files were screened resulting in with 54 patients (6.6%) with BRMs and 774 (93.4%) without BRMs. **B** Incidence of BRMs in cerebral imaging with previous clinical signs (*n* = 54). The column *no BRMs* (despite cCT/cMRI) includes patients without BRMs in cerebral imaging with clinical suspicion for brain metastases (*n* = 55). Clinical signs for brain metastases included symptoms like headache, dizziness or focal neurological symptoms. All other patients had no clinical signs and therefore no cCT/cMRI performed. **C** Incidence of BRMs in patients with initially advanced disease stadium (*n* = 245 patients, BRMs in *n* = 24 patients, no BRMs in *n* = 221 patients). **D** Incidence of BRMs in patients with initially limited disease stadium (*n* = 577 patients, BRMs in *n* = 30 patients, no BRMs in *n* = 547 patients). **E** All patients with BRMs (*n* = 54) divided into whether there initial disease stadium was limited (*n* = 20, 44.4%) or advanced (*n* = 34, 55.6%)

We identified 827 patients for further evaluation of this retrospective study (Fig. 1). All patients with neurological or other symptoms suspicious for brain metastases (BRMs) received either cMRI or cCT (*n* = 109 patients), respectively. In 54 patients (49.5%) cerebral imaging confirmed BRMs (Fig. 2B). In total, the incidence for BRMs was 6.5% (Fig. 2A).

The vast majority of GEC patients developing BRMs were male (90.7%, *p* = 0.005, RR: 3.251, 95% CI 1.365–7.863) and the median age at diagnosis of BRMs was 63.8 years (45.1–84.8 years). In patients developing BRMs, the most frequent histology was adenocarcinoma (87.5%, *p* = 0.01, RR: 2.883, 95% CI 1.213–6.971) (Table 1).

Furthermore, the primary tumor locations among patients with BRMs was evenly distributed between esophageal and stomach (Table 2). The HER2 status was positive (+ + or +  + +) in 18.5% and negative (− or +) in 37.03% of patients with BRMs and missing in 44.44% of patients.

**Table 2:** Clinical characteristics of patients with brain metastases

	Number of patients	Percent (%)
Gender (%)		
Male	49	90.7
Female	5	9.3
Strategy at initial diagnosis		
Limited disease (resectable)	30	55.6
Advanced disease (non-resectable)	24	44.4
Histology		
Adenocarcinoma	50	91.1
Squamous cell carcinoma	5	8.9
Her2-status		
Her2 pos	10	18.5
Her2 neg	20	37.0
Missing	24	44.4
Primary tumor localisation		
Eosophageal	11	20.4
Gastroesophageal junction	27	50.1
Siewert type I	11	20.4
Siewert type II	13	24.1
Siewert type III	3	5.6
Stomach	13	24.1
Overlapping	3	5.6
Initial chemotherapy		
FLOT	11	32.4
CROSS	9	26.5
CF	1	2.9
EOX	4	11.8
ECF	1	2.9
Upfront surgery	3	8.8
Treatment approach		
Upfront surgery	3	5.6
After neoadjuvant treatment	23	42.6
No surgery due to progress	3	5.6
Palliative	23	42.6
Missing	2	3.7
Cerebral metastasis at initial diagnosis		
Yes	11	20.4
No	43	79.6
BRMs as only site of metastasis (only initially resected)		
Yes	16	61.5
No	10	38.5
First contact to UMG with BRMs (only initially resected)		
Yes	5	19.2
No	21	80.7
First contact to UMG with BRMs (all patients)		
Yes	9	16.7
No	45	83.3

*Her2* human epidermal growth factor receptor 2; *FLOT* 5-fluorouracil, leucovorin, oxaliplatin, docetaxel; *CROSS* radiation (41, 4 Gy) plus carboplatin and paclitaxel; *CF* cisplatin, 5-fluorouracil; *EOX* epirubicin, oxaliplatin and capecitabine; *ECF* epirubicin, cisplatin and 5-fluouracil; *BRM* brain metastases; *UMG* University Medical Center Göttingen

All patients with brain metastases received a platinum based and 5-fluorouracil containing chemotherapy or chemoradiation in neoadjuvant, perioperative or palliative settings (Table 2).

Moreover, we correlated the incidence of BRMs with primary tumor stages at time of diagnosis, dividing the patient cohort into those with limited (potentially resectable), and advanced (irresectable and/or metastatic) GEC. The data is illustrated in Fig. 2. Of note, the incidence of BRMs in limited GEC was 5.2% (30 of 547 patients) and in advanced GEC was 9.8% (24 of 221 patients) respectively (Fig. 2C, D). Of 54 GEC patients with BRMs, at the initial diagnosis, 20 patients had a limited disease (44.4%) and 34 patients already had an advanced disease (55.6%) (Fig. 2E). The incidence of BRMs was significantly higher during disease progression in patients with advanced GEC at initial diagnosis (*p* = 0.0201) (Table 1).

Interestingly, after curative resection of the primary tumor, BRMs are frequently the only site of metastases (16 of 26, 61.5%, Table 2). To avoid any bias as a university hospital with referral of more severe cases with higher frequency of BRMs we evaluated the number of patients referred to our institution due to BRMs for the first time. We found only in a small proportion of patients (*n* = 9, 16.7% in all patients and *n* = 5, 19.2% in those with resected tumors) a first time referral due to BRMs (Table 2).

Among all patients with BRMs, 11 patients (20.4%) had BRMs at initial diagnosis while in the majority of patients (*n* = 43, 79.6%) BRMs were detected in the later course of the disease due to newly developed symptoms raising suspicion for BRMs (Table 2).

**Fig. 3:**
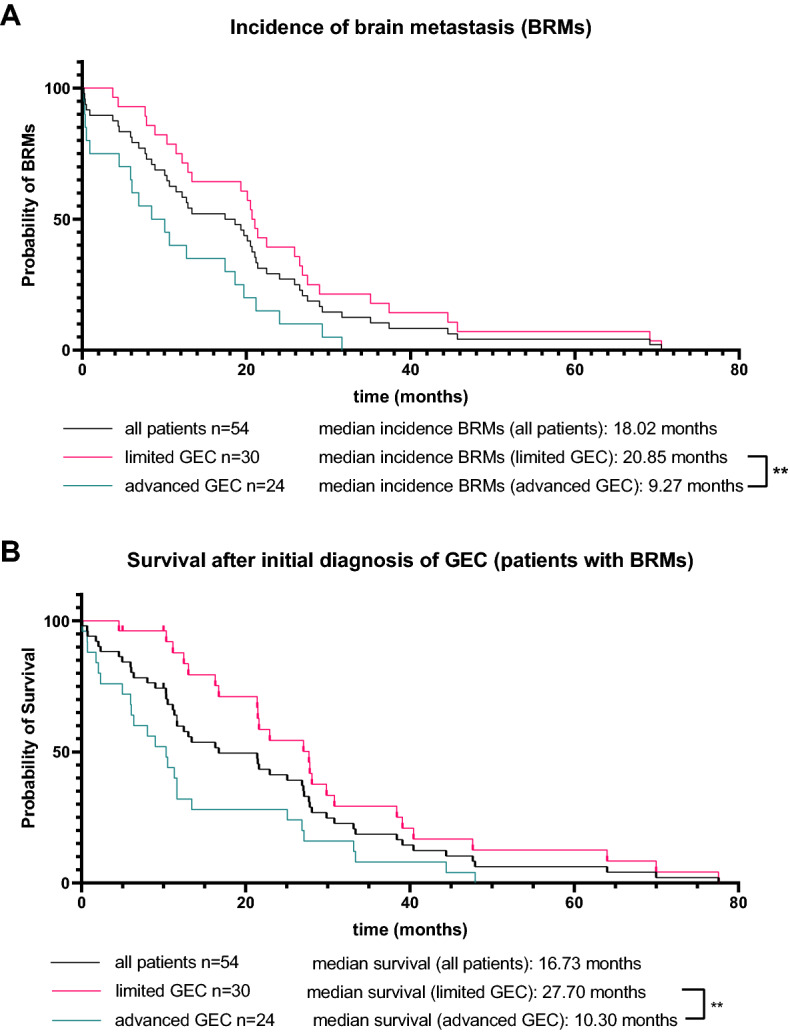
Appearance of brain metastases. **A** Appearance of brain metastases in all patients (black line, median = 18.02 months) or patients with limited GEC (red line, median = 20.85 months) and advanced GEC (red line, median = 9.27 months) from the timepoint of initial tumor diagnosis. **B** Survival of all patients (black line, median = 16.73 months) or patients with primarily limited GEC (red line, median = 27.7 months) and primarily advanced GEC (green line, median = 10.3 months)

This illustrates that BRMs usually appear in the later course of the disease. In our patient cohort, the median time from disease onset to diagnosis of BRMs was 18.02 months. Moreover, in patients with primarily resected tumors (limited disease) the time from diagnosis to appearance of BRMs was 20.85 months and in those patients with non-resectable tumors (advanced disease) 9.27 months, respectively (*p* = 0.0026, Fig. 3A).

**Fig. 4:**
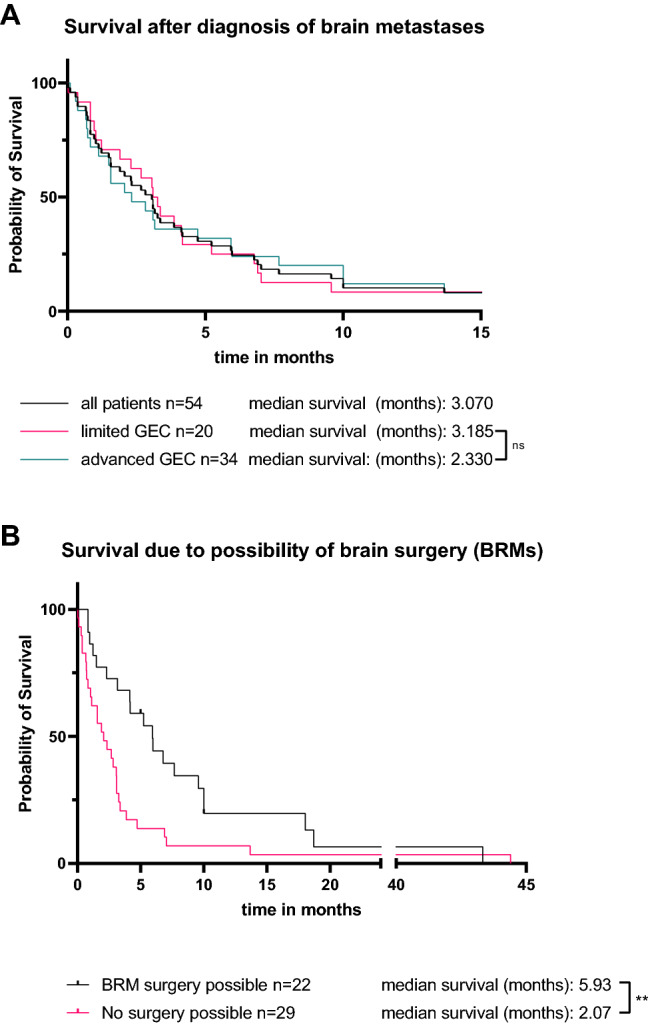
Survival of patients after diagnosis of brain metastases. **A** Survival after diagnosis of brain metastases in all patients (black line) or patients with primarily limited GEC (green line) or primarily advanced GEC (red line). **B** Survival of patients with resectable (green line) or unresectable (red line) brain metastases after diagnosis of brain metastases via cerebral imaging

Usually, the onset of BRMs represents a sign of poor prognosis. The median survival after the diagnosis of brain metastases was 3.07 months (Fig. 4A). There was no significant difference between primarily resected and non-resectable patients (3.185 months vs. 2.33 months, *p* = 0.64, Fig. 4A). However, correlating the survival after diagnosis of BRMs with the possibility of removal of BRMs with brain surgery, there is a significant difference between these groups. A significantly longer survival in patients with possible and successful brain surgery was shown (5.93 months vs. 2.07 months, *p* = 0.023, Fig. 4B), with two patients still being alive after successful brain surgery at the time of publication.

The median survival of patients with BRMs in both limited GEC and advanced GEC was 16.73 months. In detail, in patients with limited GEC (primarily resected tumors), the median survival after initial diagnosis was 27.37 months and in patients with advanced GEC only 10.3 months (*p* = 0.029, Fig. 3B).

## Discussion

The present study represents one of the largest cohort analysis aiming at the occurrence of brain metastases (BRM) in carcinomas of the esophagus and stomach (GEC). In this type of cancer, we found an incidence of BRMs of 6.52%. This incidence is slightly higher than in other similar studies with incidences ranging from 1.7% to 3.9% for esophageal and gastric carcinomas, respectively, and up to 5.9% for carcinomas of the gastroesophageal junction [13, 14, 16, 21]. Interestingly, our work showed a significantly increased risk of developing brain metastases in male patients (RR: 3.251, 95% CI 1.365–7.863). This gender specific increased incidence was similarly described in a collective of American patients (BRM in men in 3.2%, compared to 1.7% in women) [12], although this data only looked at synchronous metastases, whereas our data showed that BRM are a late event of disease progression and usually not present at initial diagnosis.

Furthermore, our analysis shows a significantly higher risk of developing brain metastases in patients with adenocarcinoma compared to squamous cell carcinoma (RR: 2.883, 95% CI 1.213–6.971). These finding are consistent with other studies, where an incidence of brain metastases in squamous cell carcinomas was less frequent (1.2–1.6%) than in adenocarcinomas, where an incidence of up to 5.9% was reported [14, 22, 23]. Interestingly, the increased incidence of brain metastases in adenocarcinomas does not occur only in GECs. In lung tumors, an incidence of brain metastases was described in 14.4% of adenocarcinomas but only in 5.3% of squamous cell carcinomas [11]. The reason why adenocarcinomas seem to have a higher likelihood of developing brain metastases is still debated. One paper demonstrated that metastatic cells from breast and lung adenocarcinomas produce neuroserpin and serpin, inhibitors of plasminogen activators, to circumvent apoptosis by astrocytes when crossing the blood–brain-barrier [24]. This phenomenon might explain why our data suggests a significantly higher incidence of BRMs in adenocarcinomas of the upper GI tract.

However, another study found a significant overlap in the genetic profile of adenocarcinomas and squamous cell carcinomas [25], indicating that the molecular changes promoting brain metastases in adenocarcinomas might be of epigenetic nature.

Furthermore, a different study suggests that the HER2 expression, naturally only present in adenocarcinomas, increases the risk for brain metastases [26]. Unfortunately, due to missing data on HER2 expression, we could analyze this correlation in our work.

However, there seems to be a correlation between the duration of tumor disease and the occurrence of brain metastases. In our cohort, the vast majority of brain metastases did not appear at the time of tumor diagnosis, even if metastases were already present in other organs. Thus, on average, it takes more than 9 months for metastases to be diagnosed in the brain with the help of cerebral imaging. As brain metastases usually develop slowly and need to have a certain size or location for them to cause neurological symptoms, the incidence we found in our data might be lower than the real incidence of brain metastases as cerebral imaging is only performed when symptoms occur. Prospective studies with routine brain imaging (MRI) have to be conducted to evaluate this question further.

We demonstrated that brain metastases frequently occur in patients who received perioperative chemotherapy or preoperative radiochemotherapy with initial curative intent. In these patients, however, the latency to the appearance of brain metastases is significantly longer, approaching 21 months. The most interesting questions arises when looking at patients who had curative surgery and developed isolated brain metastases during aftercare. To our knowledge, despite a case report, there are no studies or reviews explaining this phenomenon [27]. Possibly, cancer cells in these patients have already spread to the brain at the time of neoadjuvant chemotherapy and are not reached by standard chemotherapy agents. Consequently, this hypothesis has to be further investigated in clinical studies and could be underlined with routine MR imaging at initial diagnosis and during aftercare after successful resection.

At last, our data shows that early detection of brain metastases possibly leads to a longer survival if the brain metastases are still resectable. Our data shows a significantly prolonged median survival if brain surgery is possible (5.9 vs 2.1 months) and successfully performed. In summary, a routine cerebral imaging, both at initial diagnoses and at regular time points following initial diagnosis, could identify more asymptomatic brain metastases and therefore may prolong overall survival significantly.

## Conclusions

Our results point to a more specified implementation of cerebral imaging into routine monitoring of gastroesophageal cancer. Concisely with other studies, the brain is a frequent site of metastases in these tumors, especially in men and in adenocarcinomas. A routine cerebral MR imaging at initial diagnosis, 12–18 months after initial diagnosis in a curative situation and 6 months in a palliative situation, could identify asymptomatic brain metastases and improve treatment strategies in these patients.
